# The Effect of Vocational Training on Visually Impaired People’s Quality of Life

**DOI:** 10.3390/healthcare12060692

**Published:** 2024-03-20

**Authors:** Hui-Ying Chu, Hui-Shan Chan

**Affiliations:** 1 Department of Living Services Industry, Tainan University of Technology, Tainan City 710, Taiwan; t10032@gm.tut.edu.tw; 2 Department of Applied Cosmetology, National Tainan Junior College of Nursing, Tainan City 700, Taiwan; 3 Department of Special Education, National Tainan University, Tainan City 710, Taiwan

**Keywords:** vocational training, visually impaired, quality of life, life reconstruction, physical health

## Abstract

Background: Quality of life (QOL) is frequently utilized in clinical medicine and research to assess a patient’s health status and treatment effectiveness. Objectives: This study investigates the impact of vocational training on the QOL of visually impaired individuals. Methods: We employed the brief Taiwan version of the World Health Organization QOL Questionnaire (WHOQOL-BREFTW) to assess four domains: physical, psychological, social, and environmental, using a nonequivalent pretest–posttest control group design. The experimental group participated in 6 months of vocational training, including life and career reconstruction. After completing the vocational training, the average QOL score for the experimental group was 3.34 ± 0.18, while the control group had a score of 3.10 ± 0.85. The generalized estimating equation (GEE) results revealed a notable improvement of 10.81 (1.10) in the posttest overall QOL scores compared to the pretest scores in the control group. Conclusion: Vocational training significantly improves the overall QOL for visually impaired individuals. It is noteworthy that the psychological, social relationship, and physical health domains of WHOQOL-BREF TW exhibited the most significant improvements. This emphasizes the following: 1. professional knowledge and technical learning can enhance the abilities of the visually impaired. 2. The improvement in QOL occurs primarily at physical, psychological, and social levels. These levels involve maintaining physical health, reducing dependence on medical care, and enhancing self-care abilities for life reconstruction. 3. Integrating electronics with directional action can help to mitigate the risks associated with outdoor activities.

## 1. Introduction

In accordance with a 2021 report by the World Health Organization [1], the global population affected by vision impairment or blindness exceeds 2 billion individuals. Notably, at least 1.1 billion people contend with vision loss attributable to the absence of essential care for conditions such as myopia, hyperopia, glaucoma, and cataracts. The anticipated factors contributing to a substantial rise in these statistics include population expansion, aging demographics, and evolving lifestyles marked by diminished outdoor exposure and increased engagement in intensive near-vision activities. An alarming projection posits that, by the year 2050, an estimated half of the world’s population will grapple with vision impairment, particularly accentuated in low- and middle-income countries [2].

Preliminary estimates suggest that 10% of individuals aged 50 and above in China are poised to experience visual impairment [3]. In the United States, data derived from the 2018 National Health Interview Survey (NHIS) indicate that 23 million adults aged 18 to 64 and 9.2 million adults aged 65 and above reported significant vision loss [4]. Similarly, the United Kingdom recorded approximately 2.28 million individuals with moderate to severe vision loss in 2020, with 171,000 people classified as blind [5]. Further supporting this global trend, the Ministry of Health and Welfare of Taiwan reported an increase in visually impaired individuals from 38,747 in 2000 to 54,317 in 2022. Notably, 64.5% and 72.8% of these cases pertained to individuals aged 50 and above [6], underscoring the prevalence of visual impairment among middle-aged and older adults. Moreover, the risk of vision impairment is anticipated to escalate as the global population ages [2].

Visual impairment significantly impedes an individual’s environmental awareness given the predominant reliance on sight for perception [7]. The existing research on adaptive strategies for the visually impaired is limited, primarily focusing on the experience of vision loss [8]. Jones et al. [9] discovered that visually impaired people, in their daily lives, are more likely to experience malnourishment and a diminished quality of life. The majority of visually impaired individuals may spend many years rebuilding their careers after vision loss [9]. Sweeting et al. [10] and Aghazadeh et al. [11] underscored the extensive impact of visual impairment on activities such as reading, writing, information gathering, self-care, relationships, employment, and psychological adjustment.

A study of research papers on the mental health of visually impaired individuals by Demmin and Silverstein [12] discovered that these individuals face challenges in adjusting to their condition, leading to negative emotions such as pressure, depression, anxiety, mental fatigue, and psychological distress. Visual impairment not only involves physical and psychological challenges but also prompts a reassessment of relationships with the environment and family. Work functioning and social interactions become more demanding, with varying cognitive and practical responses among affected individuals [10,11].

People with visual impairment often experience a lower QOL due to increased risks of falls, collisions, and traffic accidents caused by poor vision. Consequently, their daily activities, employment, and independence are negatively impacted [11,13,14]. Specifically, individuals with visual impairment have fewer opportunities for leisure activities and encounter financial challenges, including a higher likelihood of financial crises due to employment difficulties [15,16]. As visual impairment becomes more prevalent in an aging society, the declining quality of life for individuals with eye disorders is an increasingly significant concern.

QOL is widely used in clinical and empirical studies to assess patients’ health and treatment effectiveness [17]. In Taiwan, the WHOQOL-BREF TW, a concise version of the World Health Organization Quality of Life Questionnaire, is commonly used to evaluate the health-related QOL of individuals, including those with severe visual impairment [18,19,20,21]. This questionnaire incorporates the 28-item Short Form Survey for physical and mental health. While previous research has explored the impact of various diseases on the QOL of people without visual impairment, there is a significant gap in understanding the QOL of visually impaired individuals. Logistical challenges in conducting experiments with this population contribute to this gap. Further research is needed to address this issue and gain insights into the QOL of visually impaired adults in Taiwan.

In an era that prioritizes holistic health, massage therapy and reflexology play vital roles in promoting healing and relaxation, especially in palliative care [22]. These alternative therapeutic modalities in the field of medicine are effective in relieving stress, enhancing overall health [23], or relieving symptoms of premenstrual syndrome (PMS) [24]. In the study by Guo et al., massage of acupoints or specific reflex zones lasting 10–20 min each time was the most effective strategy for relieving perioperative anxiety [25]. In addition, McCullough et al. found that reflexology can significantly reduce the cardiac index (CI) of healthy volunteers and salivary starch, blood pressure, and enzymes in Alzheimer’s patients [26]. The above clinical studies have shown that regular reflexology or massage therapy can significantly reduce stress, have a positive impact on anxiety levels, and improve quality of life [22,23,24,25,26].

Donoyama and Takeda [27] reported that visually impaired individuals working as massage therapists experience less fatigue and depression than those not in the profession. The study reveals mental health scores by demographics and visual impairment conditions. Visually impaired individuals with work experience scored lower on the Self-rating Depression Scale (SDS) (37.5 ± 9.7) and the State Trait Anxiety Inventory (STAI) (42.6 ± 11.7) compared to those without work experience (SDS: 40.6 ± 9.4, STAI: 46.7 ± 10.5). A *t*-test (* *p* < 0.05) indicated significantly lower depression and anxiety scores for those with work experience. This indicates that prior work experience enhances self-confidence and contributes to a calmer outlook. Furthermore, Shah, Frank, and Ehrlich [28] observed that visually impaired individuals with satisfactory employment exhibited better well-being in terms of physical health, finances, family life, and social connections compared to their unemployed counterparts. Consistent employment not only cultivates patience but also offers financial stability and opportunities for social interaction for individuals with visual impairment. In Taiwan, legal employment as a massage therapist requires possession of a Grade C technician certificate [29]. Visually impaired individuals must undergo a proficiency examination, covering both theoretical and technical assessments, to obtain a certificate. The People with Disabilities Rights Protection Act [30] requires authorities to subsidize regional agencies providing courses to help visually impaired individuals secure stable employment, promoting employment equality. Customized adaptive employment measures are essential to protect the employment rights of the visually impaired, ensuring that they receive social support, acquire job skills, and rebuild their lives, ultimately boosting self-esteem [31].

It is a common phenomenon in many countries for visually impaired people to engage in massage therapy, such as in Hong Kong [32], Vietnam [33], Australia, Ireland, India, New Zealand, Poland, the United Kingdom [34,35,36], Japan [27], and China [37]. In the UK, becoming a physiotherapist is considered a suitable profession for people who are visually impaired. Vocational physiotherapy schools provide specialized physiotherapy courses, technical learning, and career consultation [38]. Massage is defined as a medical behavior in Japan. The government actively provides opportunities for visually impaired masseurs to receive professional education [30]. In China, there are “Regulations on Medical Massage for the Blind,” which are medical practices that should be performed in medical institutions [39]. Recently, there has been increasing global attention regarding protecting the rights and interests of vulnerable and special groups. However, studies exploring the correlation between the content of visually impaired people’s vocational training and their QOL are limited. The QOL of people who experience visual impairment in their lives is a topic worth investigating. Therefore, the purpose of the present study is to explore the impact of vocational massage training on the QOL of people who are visually impaired.

## 2. Research Materials and Methods

### 2.1. Design

This study received approval from the Human Experiment and Ethics Committee of National Cheng Kung University (IRB NCKU HREC-E-107-00032). Utilizing a survey questionnaire, data were collected through in-person, phone, and online responses. We employed a quasi-experimental research design with a nonequivalent pretest–posttest control group [40]. We surveyed 173 visually impaired participants. They were categorized into (1) experimental group (n = 108) via purposive sampling; recruited comprised students from the Taiwan Rehabilitation Institute for the Blind, Yilan Muguang Reconstruction Center, and the Central District Visually Impaired Association with vocational training experience. (2) The control group (n = 65), using a random selection method, included members from various regional visually impaired associations.

A pretest was administered before the experiment, with the experimental group undergoing massage vocational training for 6 months, while the control group continued their regular activities. Following the training, a posttest was conducted to compare the quality of life (QOL) between the two groups.

### 2.2. Recruitment

The participants were recruited from Taiwan’s Institute for the Blind, the Mu-Kuang Rehabilitation Center for the Blind, and the Various regional visually Impaired Massage Therapists Union through purposive sampling. The inclusion criteria were as follows: (1) being a visually impaired person with a government-issued disability handbook (it is an official certification) and at least 20 years old; (2) not having multiple disabilities; (3) being capable of communicating and clear expression; and (4) having agreed to participate and signed an informed consent form after having received an explanation of the research objectives and content.

A pretest was conducted to analyze the research samples, utilizing data collected from 50 questionnaire respondents. G-Power was employed for estimation [41]. Based on the mean and standard deviation (3.09 ± 0.03 and 3.07 ± 0.03, respectively) of the pretest scores, an effect size of 0.59 was determined. According to Cohen, an effect size of 0.59 is considered moderate, as per his recommendation (2013) of α = 0.05 and 1 − β = 0.95. Accordingly, each group was estimated to require 62 participants, for a total of 124 participants [42].

**Figure 1:**
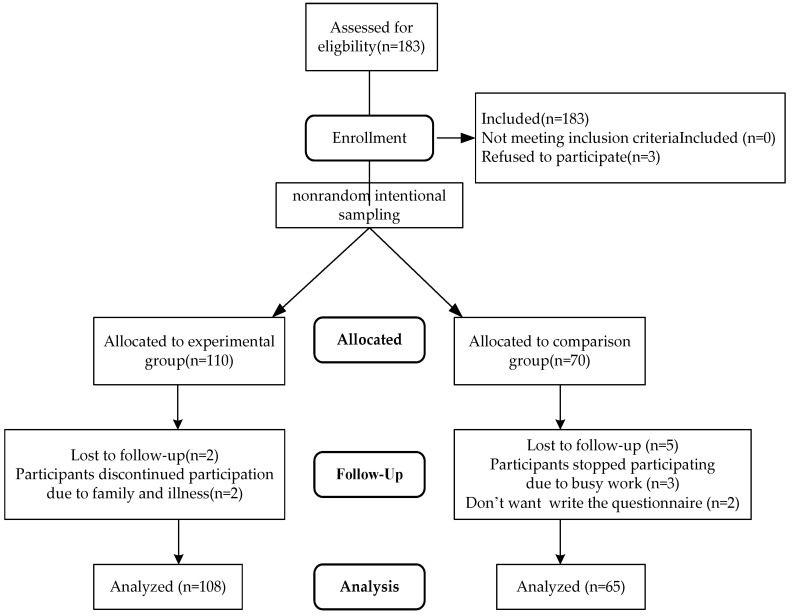
Recruitment flowchart.

A total of 183 participants met the inclusion criteria. However, three of these individuals declined to participate due to personal reasons. Of the remaining 180, 110 were assigned to the experimental group and 70 were assigned to the control group. “Two participants in the experimental group withdrew from the training course due to family matters and illness”. Five participants from the control group withdrew from the study; three did so due to their work schedules, and two did so because they were unwilling to complete the questionnaire. A total of 108 participants in the experimental group and 65 in the control group completed the study (see Figure 1).

### 2.3. Vocational Training Course for The Visually Impaired

In Taiwan, both government agencies and non-governmental organizations offer six-month vocational training courses for individuals with congenital or acquired visual impairments. Table 1 illustrates that these courses focus on four key competencies: vocational reconstruction professional skills, targeted action, life reconstruction and self-care, and information utilization. Professional competency training covers human physiology, massage technology, and workplace ethics, guiding participants toward technician certificates. Basic massage techniques consist of Effleurage, Petri sage, Friction, Tapotement, and Vibrations [25]. Effleurage involves gentle circular strokes for muscle relaxation and warming up; Friction employs circular movements to penetrate deep tissues using the thumb or a pointed object; Tapotement is rapid tapping, slapping, and cupping to strengthen deep-tissue muscles; Vibrations entail pressing and releasing tissues in an up-and-down movement.

Targeted action involves enhancing sensory perception and motor skills for independent movement. Self-care emphasizes dietary and lifestyle practices, fostering family and social development. Information utilization imparts skills in using electronic devices and online resources. These courses aim to boost confidence, abilities, and societal roles for visually impaired individuals, encouraging them to discover new roles within the community [43,44]. Life and vocational reconstruction support visually impaired individuals in acquiring essential life and career skills. These programs offer training and opportunities for social engagement, helping to establish a supportive network that enhances mental well-being and reduces isolation. Table 1 details the content provided by the Institute for the Blind of Taiwan and the Mu-Huang Rehabilitation Center for the Blind.

**Table 1:** Vocational training program for the visually impaired.

Course Units and Content
Vocational reconstruction Professional competency: Deliver human physiology and professional massage training and guide participants through the requirements the technician certificate examination to strengthen their professional competency
Introduction to human physiology, Health care Introduction to acupoints: Functions and theoretical application of the 12 meridian points
Basic massage techniques: Effleurage, Petrissage, Friction, Tapotement and Vibrations Massage.
3. Clinical massage techniques: Covering head, neck, shoulders, back, chest, abdomen, waist, buttocks, limbs, and entire body 4. Physiology and hygiene: Functions, care, and hygiene related to various organs of the body 5. Professionalism: Occupational regulations and independent skills in employment 6. Workplace internship: Occupational injury prevention; employment security and readiness
Targeted action: Train in orientation and action skills. Utilize residual vision and sensory perception to navigate the environment, and master the proper use of assistive devices for independently and safe walking.
Understanding the concepts of orientation and space and authentically feeling the physical world Developing skills in using assistive devices, such as canes, electronics, and mobile phones Sensory training, with tactile, auditory, and balance-related senses used to understand the relationship between oneself and the environment; safe walking indoors and outdoors Creating a mental map of the environment through human guidance or the hands Using public transportation
Life reconstruction and Self-care competency: Training to enable individuals with visual impaired to care for themselves in their daily lives
Cooking and using home appliances Organizing clothing and the home environment Ensuring personal hygiene and developing a daily routine Participating in leisure and social activities
Information utilization competency: Computer learning for the visually impaired to establish document processing and online information utilization skills
Using computer voice systems and expanders Using assistive devices, such as mobile phones

Source: Institute for the Blind of Taiwan [45] and Mu-Kuang Rehabilitation Center for the Blind [46].

### 2.4. Measurements

This study adopted a nonequivalent pretest–posttest control group design. We obtained demographic information and used the World Health Organization QOL Questionnaire BREF Taiwan Version (WHOQOL-BREF TW) [19].

#### 2.4.1. Demographic Information

This study collected information on each participant’s sex, age, marital status, education level, occupation, occupation category, monthly income, religious beliefs, lifestyle, independent mobility, age of vision impairment, cause of vision impairment, and their significant others.

#### 2.4.2. WHOQOL-BREF TW

The WHOQOL-BREF TW [19] comprises the following domains: physical health, including physiological health and independence (7 items); psychological factors, including psychological condition, spiritual condition, religious beliefs, and personal beliefs (6 items); social relationships (4 items); and environmental factors (9 items). The items in these domains and two general evaluation items comprise 28 total items. The items were general items scored according to the participants’ subjective feelings, and their inclusion enabled a comparison across ethnic and cultural groups. Each item was scored using a 5-point Likert scale, with a higher score indicating a higher QOL [47,48]. We calculated the score for each domain by multiplying the sum of the scores of all items within the domain by four and dividing it by the number of items in that domain, with total scores for each domain ranging from 4 to 20.

The WHOQOL-BREF TW was verified as having satisfactory reliability and validity, with an overall internal consistency value of 0.97 and a test–retest reliability value of 0.86 [20]. The reliability values of the domains related to physical health, psychological factors, social relationships, and environmental factors were 0.76, 0.70, 0.68, and 0.75, respectively. We assessed the validity of each domain through a model fitness test through confirmatory factor analysis (CFA) [47]. The comparative fit indices of the domains related to physical health, psychological factors, social relationships, and environmental factors were 0.916, 0.980, 0.998, and 0.907, respectively. The overall comparative fit index was 0.886, indicating satisfactory overall and domain fit. Three directors of rehabilitation agencies for the visually impaired, two scholars, one senior nursing professional, and one physician were invited to assess the questionnaire’s content validity. The content validity index (CVI) value was 0.84, exceeding the threshold of 0.8 [47]. This finding verified that the questionnaire is suitable for assessing the QOL of individuals with visual impairment. The *t* values of the item analysis were 3.12–15.08 (*p* < 0.01), indicating satisfactory discriminant validity. The questionnaire was formalized accordingly.

### 2.5. Statistical Analysis

We used SPSS version 20.0 (SPSS, Chicago, IL, USA) to perform the statistical analyses. A simple percentage analysis was conducted to assess the distribution of the demographic variables in terms of the means, standard deviations, and scale scores. A chi-squared test was used to test the homogeneity of the demographic variables of the experimental and control groups. An independent sample *t*-test was conducted to test the differences in the pretest results for QOL between the two groups. We employed a generalized estimating equation (GEE) model to measure the change in QOL score for the experimental group after their participating in the vocational training course; GEE statistical analysis was utilized in repeated measures research.

### 2.6. Ethical Considerations

Participants were recruited openly through their connection with institution managers, who received explanations of the research objective and methods and agreed to assist with recruitment. Interviews and survey questionnaires were conducted with the participants’ prior consent. This study was approved according to the National Cheng Kung University Governance Framework for Human Research Ethics (IRB 106-285). It was conducted in accordance with the principles of informed consent, equality, respect, and confidentiality.

## 3. Results

### 3.1. Comparing Personal Characteristics

Table 2 summarizes the characteristics of 173 visually impaired people; on average, age they are 41.5 years old, predominantly single, and possess an educational level above college. The majority follow Buddhism and live with family members. Parents and spouses are the primary companions, followed by children and siblings. Diseases account for the highest proportion of severe visual impairment or blindness at 47.9%, including cataracts, glaucoma, age-related mechanical diseases, ARMD, core activities, and diabetic retinopathy, followed by 35.3% regarding congenital blindness and 11.4% regarding accidental injuries, such as car accidents and occupational injuries.

A chi-squared test was used to analyze the categorical variables between the two groups in vocational training courses to understand whether there is a significant difference in demographic variables between the experimental and control groups. As shown in Table 2, the average age of the experimental group and the control group is 40.3 and 42.7 years old, respectively; the gender of the participants is 55.5% male and 43.5% female; an educational level above college resulted in 46.3% and 50.8%, respectively; 57.4% and 38.4% of the study subjects believe in Buddhism, respectively; the proportion of single people is 46.3% and 46.2%, respectively; and the proportion living with family members is 75.0% and 70.8%, respectively. The chi-squared test results show no significant difference in demographic variables between the two groups (*p* > 0.05), and they were highly homogeneous. Therefore, this study suggests that no difference in demographic variables between the subgroups affected the evaluation of the study purpose. An independent samples *t*-test was conducted on the pretest means of 3.09 (0.36) and 3.07 (0.32) for the two groups regarding the QOL questionnaire. The results show that the two groups had no significant difference in pretest values (*p* > 0.05). This indicates that the two subject groups were homogeneous regarding QOL.

### 3.2. Overall and QOL Questionnaire of the Visually Impaired Participants before Vocational Training

The QOL questionnaire scores ranged from 16 to 80 points, with 5 points per question in four domains: physical health, psychological, social relationships, and the environment (5 to 20 points each). Higher scores indicate better QOL. In the pretest analysis, the visually impaired participants scored an overall mean of 49.87 (7.86), the experimental group scored 49.77 (7.73), and the control group scored 49.97 (7.97). These scores were slightly lower than the ordinary adults’ score of 56 (8.53) [19]. Among the domains, social relationships had the highest score of 13.14 (3.07), followed by the environment (12.95, 2.92), psychological (12.89, 2.99), and physical health (10.82, 2.64). The overall evaluation scores for the experimental and control groups were around the median values of 3.09 (0.76) and 3.07 (0.72), indicating moderate QOL values and health status for both groups.

In terms of physical health, satisfaction with sleep (question 16) averaged 3.10 (1.06), and having enough energy for daily life (question 10) scored relatively high at 3.01 (0.93). However, satisfaction with work capacity (question 18) was relatively low at 2.32 (0.89). In the psychological domain, acceptance of bodily appearance (question 11) scored 3.61 (0.78), while experiencing negative feelings (question 26) scored relatively high at 3.42 (0.69). Enjoyment of life (question 5) was relatively low at 2.66 (0.75). Regarding social relationships, satisfaction with support from friends (question 22) and personal relationships (question 20) scored relatively high at 3.55 (1.10) and 3.38 (0.96), respectively. However, satisfaction with sex life (question 21) was relatively low at 3.11 (0.78). In the environmental domain, satisfaction with access to health services (question 24) and transport (question 25) scored relatively high at 4.16 (0.48) and 3.88 (0.97), respectively, while having enough money to meet needs (question 12) was relatively low.

An independent samples *t*-test was employed to determine if there were differences in the mean of the QOL scores across various domains between the two groups before vocational training. The results indicate no significant differences in the physical health domain (*p* = 0.23), the psychological domain (*p* = 0.79), the social relationships domain (*p* = 0.20), or the environmental domain (*p* = 0.50), except for the differences in questions 25 (How satisfied are you with your transport?) (*p* = 0.00) and 28 (Are you usually able to get what you like to eat?) (*p* = 0.00). In the environmental domain, there was no significant difference regarding SD (*p* > 0.05). The two groups were highly homogeneous.

### 3.3. Comparing Pretest and Posttest Scores of the Visually Impaired Participants’ QOL after Vocational Training

Table 3 shows the findings from the independent samples test used to understand whether there was a significant difference in the scores of the two subject groups on the QOL pretest and posttest. The results indicate that, regarding the experimental group, the total scores obtained on the pretest and posttest were 50.11 and 60.58 (*p* = 0.001), respectively. The control groups were 49.97 and 50.11 (*p* = 0.090), respectively. This indicates that the QOL score of the experimental group significantly improved.

**Figure 2:**
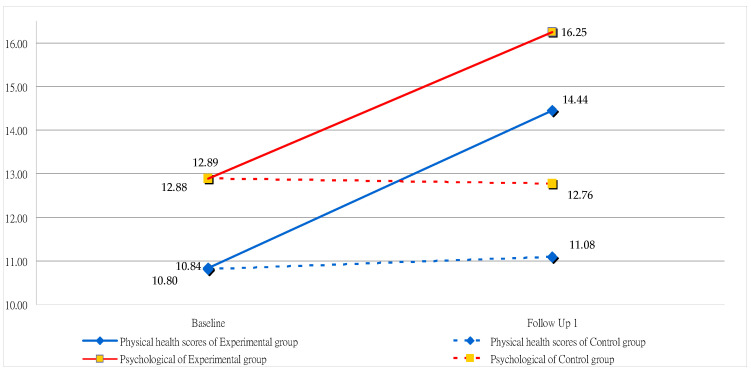
Changes in scores of the experimental and control groups for the physical health and psychological domains.

**Figure 3:**
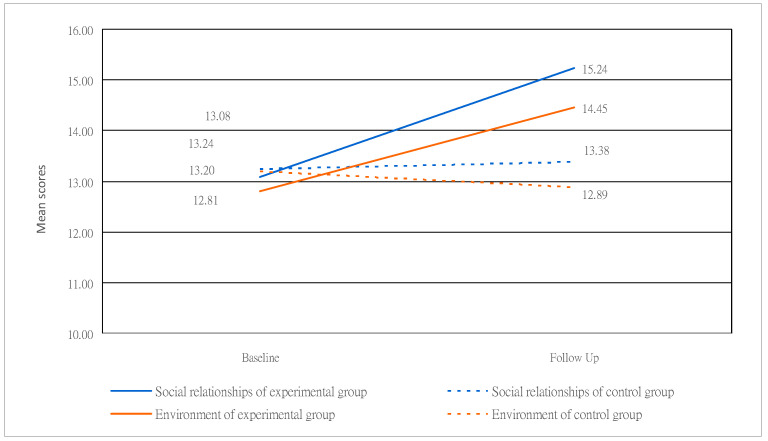
Changes in scores of the experimental and control groups for the social relationships and environmental domains.

Regarding the subjects’ posttest scores in the experimental and control groups, among the four domains, the values of the physical health domain were 14.44 and 11.08 (*p* = 0.001), respectively. Those of the psychological domain were 16.25 and 12.76 (*p* = 0.001), respectively. In the social relationships domain, they were 15.44 and 13.38 (*p* = 0.001), respectively. The environmental domains were 14.45 and 12.89 (*p* = 0.001), respectively. The above data show a significant trend regarding difference and an upward trend. This is shown in Figure 2 and Figure 3, where questions 9 (How healthy is your physical environment?) (*p* = 0.91), 23 (How satisfied are you with the conditions of your living place?) (*p* = 0.98), 24 (How satisfied are you with your access to health services?) (*p* = 0.36), 25 (How satisfied are you with your transport?) (*p* = 0.61), and 28 (Are you usually able to get the things you like to eat?) (*p* = 0.84) in the environmental domain showed no difference. The scores of the other four domains and the mean of various questions all differed significantly (*p* < 0.05).

In the posttest results for both the experimental and control groups, the psychological and social relationships domains recorded the highest means among the four domains. This suggests that the visually impaired individuals in the study enjoyed positive interpersonal relationships and received support and respect from those around them. They expressed satisfaction with life enjoyment, the meaning of life, and mental focus. Additionally, the findings indicated contentment with their living environment, transportation, daily diet, and medical and health services. In the physical health domain, severe visual impairment presented obstacles in daily activities, mobility, and workability, leading to a lower quality of life (QOL). However, after the vocational training intervention, significant improvements were observed. Physiological aspects include improvements in sleep, reduced dependence on medical care, and increased satisfaction and daily life abilities. This positive outcome was primarily attributed to the overall enhancement in QOL through the physiological health care and massage training and psychological and social relationships domains. The study’s findings affirm that the participation of visually impaired individuals in vocational training contributes to life and career reconstruction.

In the posttest for the environmental domains, no differences were found in five questions, covering aspects like the health of the physical environment (air, noise, etc.), satisfaction with transport conditions, and the ability to enjoy preferred food, etc. These questions relate to living conditions influenced by environmental factors. As a result, the vocational training intervention did not have a direct impact on these aspects. However, it was observed that, after receiving vocational training, the quality of life (QOL) scores for the experimental group exhibited a significant improvement trend, with the means of most items generally surpassing those of the control group.

### 3.4. Differences in the Scores of the Experimental and Control Groups on the QOL Questionnaire in Vocational Training

To understand the effect of vocational training, this study used the first-order auto-regressive (ARI) model of the generalized estimating equation (GEE) to process data dependency issues and estimate whether there was a significant difference between the mean of the QOL questionnaire of the experimental and control groups at different time points in the pre- and posttests. Due to the chi-squared test of demographic variables, which verified that the experimental and control groups were homogeneous in demographic variables, demographic variables were not included in the control factor. Therefore, “group” and “test time” were used for the main effects test and interaction analysis.

**Figure 4:**
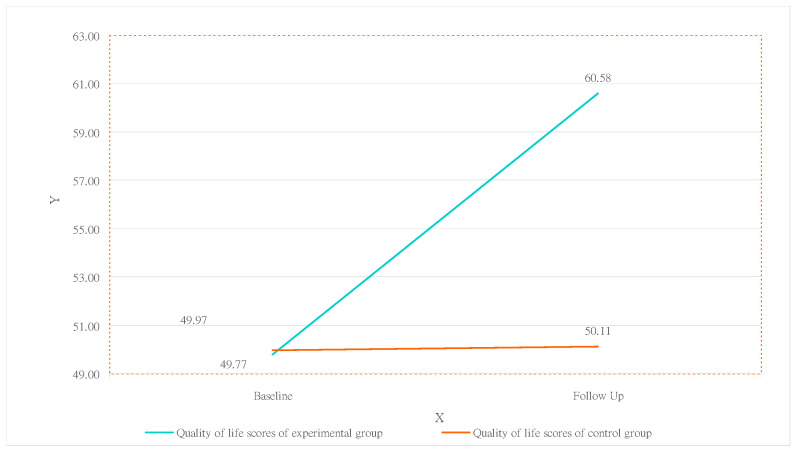
Changes in scores of the experimental and control groups regarding QOL.

As shown in Table 4, in the QOL questionnaire, the statistical results indicate that the primary effects of “group” and “test time” were not significant (*p* = 0.142 and *p* = 0.113). However, due to the significant interaction between “group” and “test time” (*p* < 0.001), this study compared the differences between the experimental and control groups under the interaction of time using the “pretest of the control group” as the benchmark value. We found that the mean of the experimental group significantly increased from 49.77 (1.79) in the pretest to 60.58 (1.15); in the control group, the score difference changed little from 49.97 (1.79) in the pretest to 50.11 (1.26). The GEE test results show that, compared to the control group pretest, the experimental group pretest improved the “QOL” by 10.81 (1.10) (*β* = 5.25, *p* < 0.001). This finding indicates that vocational training indeed had a significant effect on improving the subjects’ QOL. The above findings are summarized and shown in Table 4 and Figure 4.

## 4. Discussion

QOL reflects people’s living conditions [49]. In recent years, protecting the rights and interests of vulnerable and special groups has garnered increasing attention worldwide. Clinical Medical Care and the World Health Organization [50] have emphasized the issues of QOL related to health [51]. Therefore, this study is presented focused on the QOL of visually impaired people. Among the various physical and mental disabilities (such as hearing and physical disabilities), “severe visual impaired” is a difficult disability to overcome [52,53,54,55]. Severely visually impaired individuals have a limited number of suitable occupations, impacting their economic income and overall quality of life [56,57].

Vocational training has long been applied for visually impaired individuals [58,59]. In the pretest, both participant groups had an average quality of life (QOL) score of 49.77. After 6 months of vocational training, the experimental group’s QOL score significantly increased from 49.77 to 60.58. This study affirms that vocational training positively impacts QOL, contributing to life and career reconstruction. In psychology, such training fosters improved social interaction, concentration, and appreciation for peer support. Minimizing negative emotions, embracing life, and finding satisfaction within oneself are key aspects. Additionally, learning physiological health care and massage techniques enhances physical well-being, improves blood circulation, boosts daily activity, and reduces reliance on medical treatments. In comparing the pretest and posttest results between the experimental and control groups, the experimental group’s QOL notably improved compared to the control group, aligning with findings from previous international research [60]. Countries like the UK [38], China [39], Japan [27], Poland [61], and others have utilized vocational training to enhance QOL for visually impaired individuals.

Our research findings suggest that the QOL of people with severe visual impairment and total blindness can be improved through vocational training. The three main reasons for these overall findings are as follows:

First, training allows for the cultivation of specific professional abilities with courses to learn professional massage knowledge (e.g., human physiology, hygiene, meridians, etc.) and professional massage skills (e.g., effleurage, kneading, picking up, percussion, etc.). In particular, the experimental group participants showed significant improvement in posttest results in the domain of physical health, with the values increasing from 11.08 to 14.44 (the highest scores observed in questions 4, 10, and 17). The primary focus was on reducing dependence on medical care, enhancing daily activity abilities, and maintaining sufficient life energy. These findings are consistent with previous research [23,24,62,63]. Learning physiological massage has been proven to enhance physical health and promote blood circulation. These positive physiological changes significantly impacted the overall quality of life for the participants. In addition, learning to become a professional massage therapist is beneficial as it can help them enter the workforce and provide economic support for their lives.

Second, the psychological domain score increased from 12.88 to 16.25, and the social relationships domain score increased from 13.24 to 15.44, illustrating that learning to interact with people in groups through verbal and physical contact may enhance interpersonal relationships, fostering the development of friendships and boosting confidence. This, in turn, contributes to a more meaningful life. Relevant studies have shown that interacting with others in a friendly environment can reduce social isolation, anxiety, and loneliness [27,64,65,66], especially for participants in experimental groups who meet other visually impaired individuals during course group learning activities.

The course includes topics such as life reconstruction and the cultivation of self-care skills. It covers learning to manage daily life activities, including the ability to cook, operate household appliances, take a shower, select clothes, touch paper bills with one’s hand to distinguish between banknotes of different value, touching food and product packaging with one’s hand, and cleaning and organizing the environment. This aspect is similar to the career reconstruction service for the visually impaired in the United States, where a comprehensive assessment service provided by consultants integrates the ability to live independently into employment services [67].

Third, the training enhanced their ability to apply information, such as learning to use smartphones to take public transportation. Previous studies have found that, in the process of career reconstruction, people with visual impairment primarily require orientation and mobility training [68,69]. This is because severely visually impaired people lack basic knowledge of orientation and mobility, making it challenging to go out alone [46], often due to physical injuries such as colliding with objects or falling while walking. The difficulty of walking outside can easily lead to a psychological burden. As such, blind and visually impaired people may not want to go out and are afraid of being laughed at after falling. These conditions can be ascribed to a lack of orientation and mobility training [70] or unfriendly environmental conditions (pedestrian obstacles, a lack of warning sounds, etc.). Kuriakose et al. [71] found that using technological aids increases the possibility of employment for visually impaired people. Blind and visually impaired people can use smartphone voice guidance, which increases the convenience of going out and moving and provides more network application program information, such as GPS (Smartphone GPS Navigation) positioning and cane applications, to enhance the safety of indoor and outdoor walking.

This study used verified and effective tools to assess QOL [47,48], and several visually impaired experts and team members provided information and opinions. We used the GEE model to compare the impact of two participant groups on QOL. To our knowledge, this is the first time this technology has been used to study the experiences of visually impaired people. Our results can provide a reference for social and political institutions in reconstructing the lives of people with physical and mental disabilities. This study has limitations, including the exclusive inclusion of individuals with severe visual impairment or complete blindness, posing challenges for some participants in self-administering the questionnaire. About one third utilized 3C products and specialized computers online, while the remaining two thirds relied on paper-based questionnaires with assistance from team members collecting oral responses. Moreover, the sensitivity of two to three items in the quality of life (QOL) questionnaire regarding personal privacy may have led participants to hesitate or withhold truthful information, particularly in question 21 about satisfaction with sexual life. Another limitation is associated with the study’s focus on the impact of vocational training on the quality of life of visually impaired individuals; the participants in the experimental group were selectively recruited from visually impaired reconstruction organizations through purposive sampling, while the control group consisted of participants from regional visually impaired groups using a random selection method. Therefore, this study lacks allocation concealment, features of a randomized controlled trial (RCT), and blind outcome assessors; unable to record the intervention protocol, the researchers did not provide any treatment or intervention as is typically completed in an RCT.

Previous studies have highlighted that visually impaired people may not be able to achieve complete life adaptation without receiving intervention counseling [28,29,30,31,72]. Our results confirmed that a vocational training intervention can help to improve the effectiveness of life and career reconstruction for visually impaired people. Although we cannot infer the long-term changes in the study subjects’ QOL, conducting followups and continual tracking are necessary for government agencies to plan employment policies and help rebuild the lives of visually impaired people.

## 5. Conclusions and Suggestions

QOL-related domains have been used extensively to measure the effectiveness of services provided to individuals with physical or mental disabilities. The study concluded the following:

1. In terms of career reconstruction, a massage therapist is one of the vocational training options available for the visually impaired. Massage vocational training is designed to assist the visually impaired in acquiring professional knowledge and techniques in massage, enabling them to obtain licenses and become certified massage therapists. The employment threshold is relatively quick and easy.

2. In the realm of life reconstruction, acquiring life skills can enhance the self-care abilities of visually impaired individuals, thereby improving their quality of life. Additionally, the enhancement of psychological and social relationships comes from increasing the support within interpersonal networks and cultivating self-confidence, which can provide spiritual satisfaction to the visually impaired and reduce the negative emotions caused by helplessness.

3. Concerning the improvement in life quality, learning knowledge about physiological health care can help to maintain physical health and reduce reliance on medical treatment. Simultaneously, learning to use electronic products facilitates quick access to external information and promotes interaction with others, and directional mobility training can also reduce the risk of going out.

Based on the above, we propose the following recommendations:

1. Encourage visually impaired individuals to enhance their professional skills by participating in vocational training and seeking support from vocational and psychological counseling.

2. Establish a vocational training mechanism, including the creation of a systematic and comprehensive education system. Regularly review, track, and make necessary corrections to ensure that the training course content aligns with workplace requirements and is suitable for talent cultivation.

3. Establish dedicated government agencies to allocate fixed job vacancies for disabled individuals to work in enterprises and provide employment incentives for people with disabilities to improve quality of life.

## Figures and Tables

**Table 2:** Chi-squared test results for the experimental and control groups’ demographic variables.

Variables		All Participants (N = 173) n (%)	Experimental Group (N = 108) n (%)	Control Group (N = 65) n (%)	χ^2^	*p*
Age (years)	20–30 years old	36 (20.7%)	23 (21.3%)	13 (20.1%)	0.674	0.879
	31–40 years old	40 (22.8%)	26 (24.1%)	14 (21.5%)		
	41–50 years old	60 (34.5%)	38 (35.2%)	22 (33.8%)		
	51–60 years old	37 (22.0%)	21 (19.4%)	16 (24.6%)		
Average age		41.3	40.3	42.7		
Gender	Male	97 (55.5%)	59 (54.6%)	38 (58.5%)	0.242	0.639
	Female	76 (43.5%)	49 (45.4%)	27 (41.5%)		
Educational level	Junior and below	20 (11.7%)	12 (11.1%)	8 (12.3%)	1.233	0.749
	Senior high	34 (36.0%)	43 (39.8%)	21 (32.3%)		
	College and university	83 (48.6%)	50 (46.3%)	33 (50.8%)		
	Graduate school	6 (3.5%)	3 (2.8%)	3 (4.6%)		
Religion	None	38 (21.9%)	21 (19.4%)	17 (26.1%)	1.427	0.929
	Buddhism	89 (51.4%)	62 (57.4%)	27 (41.5%)		
	Taoism	31 (17.9%)	15 (13.8%)	16 (24.6%)		
	Christianity	15 (8.7%)	10 (9.25%)	5 (7.60%)		
Marital status	Married	74 (43.2%)	45 (41.7%)	29 (44.6%)	0.374	0.856
	Single	80 (46.3%)	50 (46.3%)	30 (46.2%)		
	Others	19 (10.6%)	13 (12.0%)	6 (9.2%)		
Visual impair reasons	Disease	83 (47.9%)	43 (24.9%)	40 (23.1%)	0.377	0.945
	Injury	29 (16.7%)	16 (9.3%)	13 (7.5%)		
	Congenital	61 (35.3%)	32 (18.5%)	29 (16.8%)		
Living status	Alone	29 (16.8%)	18 (16.7%)	11 (16.9%)	0.809	0.847
	With family members	127 (73.4%)	81 (75.0%)	46 (70.8%)		
	With relatives	8 (4.6%)	4 (3.7%)	4 (6.2%)		
	Others	9 (5.2%)	5 (4.6%)	4 (6.2%)		
Variables		Mean (SD)	Mean (SD)	Mean (SD)	t-value	*p*
Quality of life		3.08 (0.74)	3.09 (0.36)	3.07 (0.32)	−1.94	0.50

**Table 3:** Comparison of QOL scores of visually impaired individuals before and after vocational training.

Variables	Overall	T0			T1		
		Experimental Group	Control Group		Experimental Group	Control Group	
	Mean (SD)	Mean (SD)	Mean (SD)	*p*	Mean (SD)	Mean (SD)	*p*
Quality of life	49.87 (7.86)	49.77 (7.73)	49.97 (7.97)	0.15	60.58 (9.15)	50.11 (8.26)	0 .001
Overall	3.08 (0.74)	3.09 (0.76)	3.07 (0.72)	0.18	3.34 (0.81)	3.10 (0.85)	0 .001
1. How would you rate your quality of life?	3.11 (0.76)	3.12 (0.87)	3.10 (0.65)	0.27	3.43 (1.16)	3.15 (0.96)	0 .001
2. How satisfied are you with your health?	3.06 (0.73)	3.07 (0.78)	3.05 (0.68)	0.36	3.25 (0.81)	3.06 (0.65)	0 .001
Physical health	10.82 (2.64)	10.84 (2.65)	10.80 (2.63)	0.23	14.44 (2.91)	11.08 (2.78)	0.001
3. How much does physical pain hinder your essential activities?	2.43 (0.87)	2.43 (0.75)	2.42 (0.98)	0.68	2.72 (0.61)	2.52 (0.87)	0.001
4. How essential is medical treatment for your daily functioning?	2.92 (0.89)	2.92 (0.84)	2.92 (0.93)	0.20	4.01 (0.88)	2.95 (0.83)	0.001
10. Do you have enough energy for everyday life?	3.03 (0.93)	3.04 (0.92)	3.01 (0.93)	0.11	3.72 (1.03)	3.05 (0.84)	0.001
15. How well are you able to get around?	2.68 (0.77)	2.70 (0.81)	2.65 (0.73)	0.09	3.42 (1.06)	2.89 (1.01)	0.001
16. How satisfied are you with your sleep?	3.08 (1.02)	3.07 (0.98)	3.10 (1.06)	0.41	3.47 (1.07)	3.25 (0.98)	0.001
17. How content are you with your daily living capabilities?	2.56 (0.68)	2.57 (0.69)	2.54 (0.67)	0.46	3.83 (1.04)	2.51 (0.64)	0.001
18. How satisfied are you with your work capacity?	2.30 (0.81)	2.28 (0.73)	2.32 (0.89)	0.34	4.10 (0.61)	2.25 (0.72)	0 .001
Psychological	12.89 (2.99)	12.88 (2.98)	12.89 (2.01)	0.79	16.25 (2.93)	12.76 (2.97)	0 .001
5. How much do you enjoy life?	2.64 (0.72)	2.62 (0.69)	2.66 (0.75)	0.32	4.09 (0.73)	2.65 (0.88)	0 .001
6. To what extent do you feel your life is meaningful?	3.17 (0.31)	3.21 (0.35)	3.12 (0.26)	0.28	4.32 (0.58)	3.25 (0.79)	0 .001
7. How well are you able to concentrate?	3.20 (0.40)	3.21 (0.15)	3.18 (0.65)	0.26	3.58 (0.56)	3.26 (0.64)	0 .001
11. Are you able to accept your bodily appearance?	3.56 (0.73)	3.51 (0.68)	3.61 (0.78)	0.12	4.18 (0.70)	3.52 (0.68)	0 .001
19. How satisfied are you with yourself?	3.32 (0.41)	3.31 (0.46)	3.32 (.035)	0.42	4.15 (0.51)	3.21 (0.53)	0 .001
26. How often do you have negative feelings such as blue mood, despair, anxiety, and depression?	3.45 (0.68)	3.48 (0.67)	3.42 (0.69)	0.21	3.97 (0.84)	3.25 (0.81)	0 .001
Social relationships	13.14 (3.07)	13.24 (3.08)	13.20 (3.05)	0.20	15.44 (3.55)	13.38 (3.45)	0 .001
20. How satisfied are you with your personal relationships?	3.37 (0.91)	3.36 (0.85)	3.38 (0.96)	0.51	3.98 (0.83)	3.35 (0.74)	0 .001
21. How satisfied are you with your sex life?	3.12 (0.82)	3.12 (0.86)	3.11 (0.78)	00.28	3.65 (0.91)	3.21 (0.65)	0 .001
22. How content are you with the support from your friends?	3.55 (1.10)	3.55 (1.12)	3.55 (1.08)	0.85	4.15 (0.45)	3.52 (0.31)	0 .001
27. Do you feel respected by others?	3.20 (0.88)	3.24 (0.91)	3.17 (0.84)	0.19	3.75 (0.68)	3.21 (0.97)	0 .001
Environment	12.95 (2.92)	12.81 (2.87)	13.08 (2.96)	0.50	14.45 (3.42)	12.89 (2.63)	0 .001
8. How safe do you feel in your daily life?	2.95 (0.51)	2.95 (0.53)	2.95 (0.49)	0.89	3.51 (0.60)	2.65 (0.95)	0 .001
9. How healthy is your physical environment?	3.17 (1.05)	3.23 (1.09)	3.11 (1.01)	0.11	3.13 (0.71)	3.10 (0.89)	0.91
12. Have you enough money to meet your needs?	2.42 (0.68)	2.42 (0.64)	2.41 (0.73)	0.58	3.28 (0.76)	3.12 (0.97)	0 .001
13. How available to you is the information that you need in your day-to-day life?	2.99 (0.95)	3.02 (0.98)	2.85 (0.92)	0.06	3.66 (0.57)	3.10 (0.53)	0 .001
14. To what extent do you have the opportunity for leisure activities?	2.71 (0.83)	2.77 (0.98)	2.65 (0.68)	0.05	3.56 (0.81)	2.42 (0.85)	0 .001
23. How satisfied are you with the conditions of your living place?	3.57 (1.11)	3.56 (1.05)	3.58 (1.16)	0.17	3.56 (0.73)	3.55 (0.61)	0.98
24. How satisfied are you with your access to health services?	4.16 (0.48)	4.16 (1.17)	4.15 (1.18)	0.13	4.15 (0.46)	4.25 (0.75)	0.36
25. How satisfied are you with your transport?	3.67 (0.97)	3.45 (0.99)	3.88 (0.97)	0.00	3.79 (0.72)	3.86 (0.78)	0.61
28. Are you usually able to get the things you like to eat?	3.63 (0.74)	3.28 (0.63)	3.97 (0.88)	0.00	3.91 (0.59)	3.99 (0.64)	0.84

T0: QOL scale score before vocational training; T1: QOL scale score after vocational training.

**Table 4:** GEE analysis of differences in outcome variables within and between the experimental and control groups.

Quality of Life					
Variable	Mean (SD)	*β*	SE	Wald *χ*^2^	*p*-Value
Intercept		57.40	1.56	273.98	<0.001
Group (EG vs. CG)		3.45	2.39	25.46	0.142
Time overall (T1 vs. T0)		−1.21	1.01	28.39	0.113
EG at T1	60.58 (1.15)				
EG at T0	49.77 (1.73)				
CG at T1	50.11 (1.26)				
CG at T0	49.97 (1.79)				
EG at T1 vs. EG at T0	10.81 (1.10)				<0.001
CG at T1 vs. CG at T0	0.14 (0.02)				0.601
Time * Group overall					
EG * (T1 vs. T0) vs. CG * (T1 vs. T0)		5.245	1.43	184.483	<0.001

Note. GEE = generalized estimating equation; T0 = baseline; T1 = 6–9 months after completing the intervention; EG = the experimental group; CG = the control group. * Interacting effects.

## Data Availability

The data presented in this study are available upon request from the corresponding author. The data are not publicly available due to ethical restrictions.
